# Nanoparticle-Doped Antibacterial and Antifungal Coatings

**DOI:** 10.3390/polym17020247

**Published:** 2025-01-20

**Authors:** Devyani Thapliyal, George D. Verros, Raj Kumar Arya

**Affiliations:** 1 Department of Chemical Engineering, Dr B R Ambedkar National Institute of Technology, Jalandhar 144011, Punjab, India; devyanithapliyal5@gmail.com; 2 Department of Chemistry, Aristotle University of Thessaloniki, Plagiari Thes., P.O. Box 454, 57500 Epanomi, Greece; gdverros@gmail.com

**Keywords:** nanofillers, antimicrobial coatings, nanoparticles, antibacterial coatings, healthcare, packaging

## Abstract

Antimicrobial polymeric coatings rely not only on their surface functionalities but also on nanoparticles (NPs). Antimicrobial coatings gain their properties from the addition of NPs into a polymeric matrix. NPs that have been used include metal-based NPs, metal oxide NPs, carbon-based nanomaterials, and organic NPs. Copper NPs and silver NPs exhibit antibacterial and antifungal properties. So, when present in coatings, they will release metal ions with the combined effect of having bacteriostatic/bactericidal properties, preventing the growth of pathogens on surfaces covered by these nano-enhanced films. In addition, metal oxide NPs such as titanium dioxide NPs (TiO_2_ NPs) and zinc oxide NPs (ZnONPs) are used as NPs in antimicrobial polymeric coatings. Under UV irradiation, these NPs show photocatalytic properties that lead to the production of reactive oxygen species (ROS) when exposed to UV radiation. After various forms of nano-carbon materials were successfully developed over the past decade, they and their derivatives from graphite/nanotubes, and composite sheets have been receiving more attention because they share an extremely large surface area, excellent mechanical strength, etc. These NPs not only show the ability to cause oxidative stress but also have the ability to release antimicrobial chemicals under control, resulting in long-lasting antibacterial action. The effectiveness and life spans of the antifouling performance of a variety of polymeric materials have been improved by adding nano-sized particles to those coatings.

## 1. Introduction

It is crucial to apply antimicrobial polymeric coatings to the surfaces of equipment and devices that do not have an interaction between food and the environment. The objective of this antimicrobial packaging is to preserve food quality, ensure safety, and increase shelf life [1]. Varied methods of creating biodegradable and secure antimicrobial packaging have been explored. These include the use of polymers harboring antimicrobial activity within their structure and the addition of selected antimicrobials. Antimicrobial polymeric coatings offer another major benefit: they provide durable defense, suited to situations where it is difficult to wash and sanitize material on a routine basis [2]. Some antimicrobial NPs have effects against fungi as well as bacteria; that is to say, they restrain or kill fungal species. Nanotechnology in polymer enhancement improves product shelf life, antibacterials added, and the advantage of sniffing out the incipient changes leading to decay. Thus, with nanotechnology packaging, extended product shelf life in addition to the presence of antibacterial agents and degradation symptoms can all be anticipated [3,4].

The phrase “nanotechnology” was coined by Richard Feynman in 1959 [5]. Due to their higher aspect ratio and larger surface area, nanocomposites—which contain at least one phase with nanometer-scale dimensions—offer better characteristics [6]. Nanocomposite films can improve functional qualities by adding NPs to the polymer matrix. NPs can act as both reinforcement for the films and active substances [7]. The entire behavior of these NPs depends on their uniform dispersion inside the polymer matrix. Abrasion resistance, corrosion and chemical resistance, wear resistance, optical performance, antireflection, flame retardancy, electrical properties, mechanical properties, barrier properties, permeability, and antibacterial and antifungal ability are just a few of the properties that can be improved by adding nanomaterials to polymer matrices [8,9,10]. Silver is used in consumer antimicrobial goods in the building, textile, cosmetic, appliance, health, and environmental industries. Numerous items that contain AgNPs have received approval from the U.S. Food and Drug Administration (FDA) [11]. The FDA has approved the commercial release of several gold nanoparticle-based diagnostic tools, and testing of other formulations is still ongoing [12].

NPs play a crucial role in enhancing the antimicrobial properties of polymeric coatings through mechanisms such as ion release, reactive oxygen species (ROS) generation, physical disruption, and surface modification.

Nanoproducts can be made from metal NPs, metal oxide NPs, carbon-based nanomaterials, and organic micro and nanomaterials. All these differences among and within different classes of nanoparticles affect their suitability for specific antimicrobial applications. In addition, in this review, the effect of nanoparticle characteristics, such as size, bricks content, and shape, on the overall antibacterial efficiency of coatings has been analyzed. New coating materials and their applications in construction, textiles, food packaging, and healthcare were highlighted in this review. The review concludes with a discussion of the problems now facing this field of study, its prospects for the future, and possible future directions.

## 2. Types of Nanoparticles

There are four types of NPs: clay, organic, inorganic, and carbon-based NPs [7]. Table 1 comprehensively compares various NPs, detailing their key properties, antimicrobial mechanisms, synthesis methods, applications, advantages, and limitations.

### 2.1. Clay and Organic NPs

In sheets or cylindrical nanotubes, natural clays like montmorillonite and halloysite are frequently employed as nanoclays [32]. They have minerals that are fine-grained and have high mechanical characteristics. Due to their biocompatibility, biodegradability, and distinctive mechanical properties, biopolymer nanofibrils like cellulose, collagen, and chitin are also frequently used [33,34]. Nanocrystalline cellulose, also known as cellulose nanocrystals, nano-fibrillated cellulose, and bacterial nanocellulose are the three main types of nanocellulose [35,36]. Due to their substantial surface area, biocompatibility, lack of toxicity, and capacity for forming films, chitosan and chitin NPs are desirable NPs. Chitosan NPs, however, encounter stability and solubility issues that structural alterations and optimization can resolve [37,38,39]. These organic NPs encapsulate hydrophobic medicines in various biopolymer films.

### 2.2. Inorganic NPs

There are two types of inorganic NPs: metal-based and metal oxide-based.

#### 2.2.1. Metal-Based NPs

CuNPs possess antimicrobial properties due to their ability to release copper ions, which have a high redox potential capable of damaging microbial cell components. These NPs can be utilized for antimicrobial activity [40,41].

AgNPs are widely used for potent antimicrobial applications. Typically synthesized through chemical reduction or green synthesis methods, AgNPs release silver ions that interact with microbial cell membranes, causing structural damage and disrupting vital enzymatic functions [16]. They are commonly incorporated into medical device coatings to prevent biofilm formation and in textiles for durable antimicrobial treatments [15]. A study by Rai et al. demonstrated their effective use in wound dressings, significantly reducing infection rates [14]. AgNPs have garnered attention in wound healing applications due to their favorable physicochemical and biological properties [42]. They are also used in food packaging and medical applications [43,44,45].

Selenium NPs offer reduced toxicity compared to elemental selenium. They can serve as a drug delivery platform, targeting specific destinations within the body [46,47,48]. Gold NPs (AuNPs) have been used in various applications, including treating gum disorders, dental caries, tissue engineering, dental implantology, and cancer diagnostics. These applications are possible due to the unique nanostructures, high surface area, and biocompatibility of AuNPs [49].

#### 2.2.2. Metal Oxide NPs

Metal oxide NPs [50], including ZnO [51,52,53,54], TiO_2_ [55,56,57], silica [58,59], aluminum oxide [60,61], iron oxide [62], and copper oxide [63], are incorporated into polymer films as active NPs. Fe_3_O_4_ NPs exhibited strong antibacterial activity against Gram-positive and Gram-negative bacteria [64]. These metal-based NPs demonstrate diverse properties and find applications in antimicrobial activity, wound healing, drug delivery, and diagnostics. TiO_2_ and ZnO NPs exhibit photocatalytic antibacterial properties. When exposed to UV light, they generate reactive oxygen species (ROS) that have antibacterial effects [7].

NPs of CuO not only have antimicrobial, antibacterial, and antioxidant activity but also serve as a UV blocker [65]. Indeed, metal oxide NPs offer a variety of applications, including being able to enhance polymer film characteristics and add antibacterial, microbial enemy-smashing functions and UV light-blocking capability. These metal oxide NPs are utilized in polymer films to improve their properties and provide additional functionalities such as antibacterial and UV-blocking effects.

ZnO NPs are known for their photocatalytic properties, which facilitate the generation of ROS under UV light, leading to microbial inactivation through oxidative stress. These are synthesized via methods like sol–gel, hydrothermal, and vapor-phase techniques. ZnO-NPs are used in coatings for medical instruments and food packaging to enhance antimicrobial protection [21,22]. For instance, Raghupathi et al. highlighted their efficacy in antimicrobial coatings for surgical masks, providing enhanced protection against pathogens [20].

### 2.3. Carbon NPs

The distinctive characteristics of carbon dots (CDs), including their low cost, high water solubility, bioactivity, minimal danger, and light absorption, have made them a viable nanomaterial for antimicrobial food packaging [66]. CDs break down and condense genomic DNA through cytoplasmic leakage, the disintegration of cell structures, and generating ROS [67]. Alas et al. [68] used solvent casting to embed CDs in PVA, resulting in flexible films with potent fluorescence and UV-blocking properties. Together with notable antibacterial activity against both Gram-positive and Gram-negative bacteria, enhanced thermal characteristics and modest antioxidant and metal-chelating properties were all displayed by the CD/PVA films.

Ezati et al. [69] synthesized pectin/gelatin-based bioactive food packaging films using sulfur-functionalized turmeric-derived CDs. The CD-added coatings enhanced mechanical qualities, water vapor permeability, and hydrophobicity while offering strong UV protection without noticeably changing transparency. Sulfur functionalized turmeric-derived -CD-containing films showed strong antibacterial activity against *L. monocytogenes* and *E. coli*, indicating their suitability for active food packaging to improve food safety and prolong shelf life.

In order to enhance the preservation quality and suppress microbes in freshly chopped cucumber, Fan et al. [70] added CDs derived from kelp to a chitosan coating solution at different CD concentrations (0%, 1.5%, 3%, and 4.5%). The findings demonstrated that CD/CH coatings improved the inhibitory zones against *E. coli* and *S. aureus* as CD concentrations increased. During storage, the cucumbers with the 4.5% CDs/CH coating had less water mobility, lessened weight loss, less firmness loss, and less ascorbic acid and flavor degradation.

Graphene-based materials, including graphene and graphene oxide (GO), have gained attention for their excellent mechanical properties, substantial electron mobility, and larger surface area than carbon-based nanomaterials [71,72]. GO is a promising nanoparticle due to its reduced tendency to agglomerate [73]. Monolayer graphene’s remarkable properties, such as its high Young’s modulus (~1000 GPa), high fracture strength (~125 GPa), excellent electrical and thermal conductivity (~100 S/cm and ~5000 W/mK, respectively), rapid charge carrier mobility (~200,000 cm^2^/Vs), and large specific surface area (theoretically calculated value, 2630 m^2^/g), are due to its distinctive structure and geometry [71]. GO exhibits antimicrobial activity primarily through physical disruption of microbial membranes and oxidative stress [28]. Synthesized using methods like the Hummers method and thermal exfoliation, GO has applications in water treatment and electronic coatings. Its large surface area and high conductivity make it suitable for diverse antimicrobial applications [74]. Research by Akhavan and Ghaderi demonstrated its effectiveness in antimicrobial coatings for water purification systems [27].

## 3. NPs with Antifungal Activity

AgNPs, ZnO NPs, and CuO NPs have been shown to be antifungal [75,76]. These NPs can attack the fungal cell wall, disrupt its activities, and by generating reactive oxygen species are similarly able to thwart bacteria. Studies have shown that NPs like AgNPs can inhibit fungal species such as *Candida* and *Aspergillus* by disrupting their cell membranes and metabolic pathways. Antifungal activity of (acrylamide/chitosan)-AgNPs hydrogel nanocomposites showed efficient microbial inhibition activity against *C. albicans* [77]. Pinchuk et al. [78] used a microwave-assisted hydrothermal process to create hydroxyapatite doped with silver and silicate-substituted hydroxyapatite doped with AgNPs. The antifungal activity of HAp doped with 1 mol% Ag^+^ ions and Si-HAp doped with 1 mol% Ag^+^ ions nanosized powders was shown to be stronger against reference strains of *C. albicans*, *C. kruzei*, and *C. tropicalis*.

Adding antifungal NPs to coatings helps to keep the product from growing mold. This can be especially important in food packaging, where the quality of the products must be maintained for longer periods [79,80]. It was found that the antifungal activity of these coatings was beneficial to preserving the quality of the goods packaged and increased their safety for consumption.

Microwave solvothermal synthesis (MSS) was used to create ZnO-Ag nanoparticles in weight percentages of 1% and 2.5% [81]. When examined utilizing minimum inhibitory concentration (MIC) analysis, the antifungal activity of 1% Ag nanoparticles and the PMMA-2.5% (ZnO-1% Ag) nanocomposite against *Candida albicans* was demonstrated. Haiouani [82] used extracts from *Thymus capitatus* and cloves to synthesize hexagonal ZnO NPs. The ZnO NPs’ antibacterial and antioxidant qualities were greatly enhanced by the application of both extracts. With a 35 mm inhibitory zone at the same dose, they demonstrated strong antifungal efficacy against *Candida albicans*.

Using a microdilution approach, the antifungal activity of silver and gold nanoparticles made from *P. harmala* leaf extracts against *C. albicon*, *A. niger*, and *Penicillium notatum* is examined [83]. The produced gold nanoparticle of *P. harmala* leaf extracts showed a zone of inhibition of 31, 26, and 28 mm, respectively. Likewise, the zones of inhibition for silver nanoparticles were 26, 30, and 36 mm, respectively. Using *Feijoa sellowiana*, Fazli et al. [84] examined the antifungal effectiveness of toothpaste for kids that contains biosynthesized silver nanoparticles (AgNPs). The antifungal efficacy of the toothpaste containing AgNPs against *Candida albicans* was significantly higher (*p* < 0.014).

Moreover, incorporating antifungal NPs helps maintain the coatings’ structural and functional integrity by preventing fungal-induced degradation.

## 4. Techniques for Incorporating NPs into Polymeric Coatings

**Figure 1:**
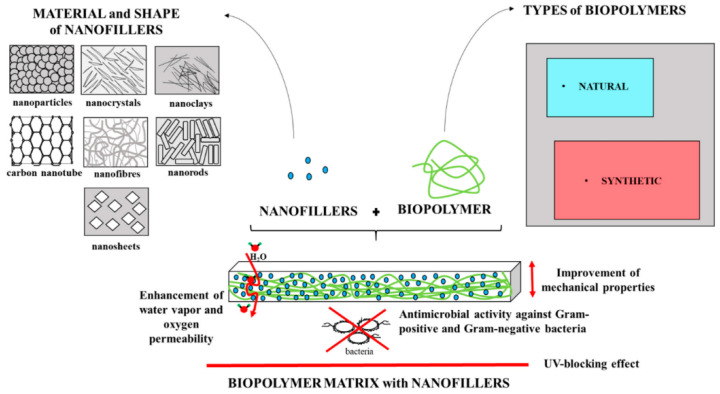
Schematic preparation of nanocomposite films and their functional properties [7].

Researchers use various techniques and technologies adapted from the chemical and pharmaceutical industries to produce encapsulated ingredients. The applications of NPs in a biopolymer matrix are shown in Figure 1. Cheap inorganic materials improve the qualities of composites and polymers. Polymer-assisted manufacturing is an efficient method for creating NPs, which uses forces to keep NPs stable. Making nanocomposites involves dispersion in a polymeric matrix utilizing techniques like melt intercalation and sol–gel technology [85].

### 4.1. Solvent-Assisted Deposition of Polymer Coatings

#### 4.1.1. Solvent Casting

The solvent casting method is a widely used method for the preparation of films containing nanocomposite scaffolds. It has several benefits including low cost and less preparation time. This method works on the principle that the polymer gets dissolved in the solvent that contains finely distributed salt particles; then the solution is placed into a predefined 3D mold. After the evaporation of the solvent, a matrix with uniformly deposited salt particles is formed. The matrix is then dipped in water so that salt particles are leached out creating uniform pores in which the desired NPs or cells can be filled [86]. This technique works well for making coatings and thin films. Only polymers that are soluble in volatile solvents can be used with this approach. Tissue scaffolds, medication delivery systems, protective coatings, and other applications are among its many uses [87,88,89,90].

#### 4.1.2. Spray Drying

Spray drying is another technique that involves atomizing a polymer solution containing dispersed NPs into tiny droplets [91]. These droplets dry into a fine powder with evenly distributed NPs embedded in the polymer matrix in a heated chamber. There are two parts to this method: spraying and drying [92]. Since this approach requires exposure to high temperatures for a shorter period than flash drying, it is also employed for drying from solutions or suspensions containing thermosensitive material [93]. The method is suitable for temperature-sensitive materials due to rapid drying. The method has several applications including in the food industry and drug delivery system. The method is limited to low-viscosity polymer solutions. A popular technique for creating a range of regulated particle sizes in food and cosmetics applications as well as pharmaceuticals is spray drying. Agglomerated powders with cohesive properties, such as flowable particles, reconstitution behavior, bulk density, and mechanical stability, can be produced by combining formulation and spray-drying conditions. It is a method for creating nanolipid powders that is both scalable and profitable. The stability, encapsulation effectiveness, shape, and particle size of nanolipid powders are all significantly impacted by this technique [94,95,96].

### 4.2. In Situ Polymerization

#### 4.2.1. Bulk Polymerization

The process that uses solution polymerization is called bulk polymerization. To obtain tiny particles, the resultant bulk polymer is mechanically ground after polymerization. To acquire the desired particle size, severing is conducted. The monomer undergoes internal bulk polymerization. Additives like initiator and transfer agents (RAFT agents) work as catalysts for the process when heat or light is applied. The molecular weight distribution of the polymer produced by this process can be readily altered by using a chain transfer agent, despite the fact that the polymer’s molecular mass distribution is typically not uniform. This method is commonly used for producing thermosetting resins, fiber-reinforced composites, and high-performance materials, where high particle content and uniform distribution are essential [97].

#### 4.2.2. Solvent-Assisted Polymerization

Solution polymerization is a technique in which monomers use a solvent as a heat sink. The selected solvent must avoid chain transfer reactions that may limit the growth of the polymer. The monomer, initiator, and resulting polymer should all be soluble in the selected solvent or solvent blend. In this technique, after the evaporation of the solvent composite is produced [98]. The method has reduced the risk of uncontrolled reactions. The method is commonly used to make adhesives and coatings [99,100]. However, the method is limited due to its environmental concerns regarding solvent usage.

#### 4.2.3. Emulsion Polymerization

Another technique to produce monodisperse polymer particles in a heterogeneous medium is emulsion polymerization. This uses an emulsion of the monomer that is generally dissolved in water in conjunction with an initiator that dissolves in water. The polymerization of the emulsion results in a polymer matrix with irregular particles [101]. Due to its water-based dispersion, the technology is environmentally safe and scalable for industrial use. Because of its controllable particle size, it has been utilized to produce latex paints and adhesives [102]. The concentration of the emulsifier must be precisely controlled for the process to work.

### 4.3. Spinning Processes

#### 4.3.1. Melt Spinning

The most affordable spinning technique is melt spinning since it does not require evaporating any solvent. This process is applied to easily melted polymers. A chamber is filled with a viscous polymer melt that is applied through a spinneret with several holes. After this, the surface of the fibers is then exposed to a blast of cold gas or air. The dispersed particles or NPs become lodged in the polymer matrix, helping to preserve their even distribution along the fiber’s length. This method has the ability to produce high-strength, high-performance fibers [103]. For thermoplastic polymers, the technique is economical. It is employed in the production of industrial fibers and textiles [104,105]. Because the required NPs are melt-spun with the polymer, the process is more effective.

#### 4.3.2. Electrospinning

Electrospinning is another method for 2D or 3D nanostructures. The principle of this method includes charge polymeric solutions, containing metallic precursors or ceramic particles with a high voltage, and the charged solution is drawn by an electric field from a nozzle onto a collector to form desirable structures. This method can be applied to produce composite materials used in sensors, tissue engineering, filtration, and energy storage applications [106]. It is a flexible, extensively researched, and often used method for creating nanofibers with unique properties like a high surface area-to-volume ratio, high porosity, adjustable characteristics, and an economical manufacturing procedure. Combining several polymers with distinct functions in the solution phase is the most effective method of creating nanofibers. The applied voltage, polymer flow rate, solution concentration, molecular weight of the polymer, relative humidity, needle diameter, and tip-to-collector distance were among the processing and polymer solution spinning parameters that affected the morphology of electrospun fiber mats [107,108]. This method has applications in the biomedical industry also [109].

#### 4.3.3. Centrifugal Spinning

Centrifugal spinning is a productive method for creating nanofibers. Using this technique, the liquid raw material is shot out of the spinning head, and the jet stretches before landing on the collector to create solidified nanofibers when the centrifugal force exceeds the substance’s surface tension. This method’s ability to regulate the fiber’s diameter and properties through spinning parameters is a significant advantage [110]. Polymers that cannot be spun by electrospinning can be spun by centrifugal spinning [111]. It is an inexpensive, simple-to-implement configuration with a minimal number of production processes. When it comes to charge-absent polymers, centrifugal spinning eliminates the need for a direct electric field, which would otherwise restrict options. Centrifugal spinning’s capacity for industrial scale-up and high production rate may be its greatest advantage over competing technologies [112].

### 4.4. Pad–Dry–Cure Method: Enhancing Nanoparticle Integration into Textiles

Textile substrates are filled with NPs using the pad–dry–cure process, which improves a number of qualities as a result, including conductivity and antibacterial activity. Using this method, iron and zinc oxide NPs have been successfully incorporated into cotton textiles and fibers, producing functionalized textiles with antibacterial and magnetic qualities [113,114,115,116]. Although the technique increases surface functionality, it is restricted to particular substrate materials and necessitates close process control. This method has several applications in textile industries [117].

### 4.5. Exfoliation–Adsorption: Creating Ordered Nanocomposite Structures

The exfoliation–adsorption method includes dispersing single layers of stacked particle materials in a polymer solution. This method decreases the gas diffusion rates in polymeric coatings. This technique is especially helpful for forming multi-layered structures that greatly improve the gas barrier qualities of coatings [118,119]. The process is complicated and demands exact control over exfoliation and adsorption, but the benefits include the creation of highly ordered structures and improved barrier qualities.

## 5. Antimicrobial Mechanisms of Nanomaterials

**Figure 2:**
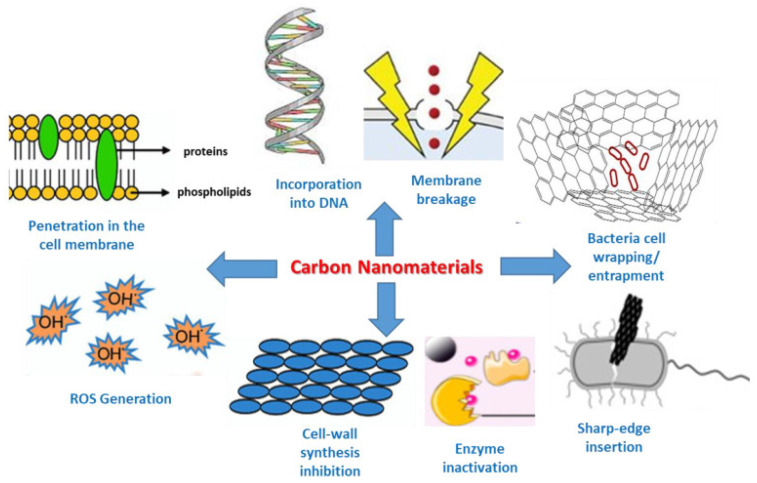
Several antimicrobial mechanisms are shown by carbon nanomaterials [121].

Nanomaterials have emerged as promising antimicrobial agents due to their unique physicochemical properties at the nanoscale. This section discusses the diverse antimicrobial mechanisms exhibited by various nanomaterials. NPs offer a variety of antibacterial activity mechanisms, including physical impairment, photocatalytic effects, oxidative stress, lipid extraction, wrapping isolation, and synergistic effects when combined with other antibacterial materials [120]. Several antimicrobial mechanisms are shown in Figure 2 by carbon nanomaterials. These nanomaterials leverage different mechanisms to combat bacterial cells, as described in the following subsections.

### 5.1. Reactive Oxygen Species (ROS) Generation

**Figure 3:**
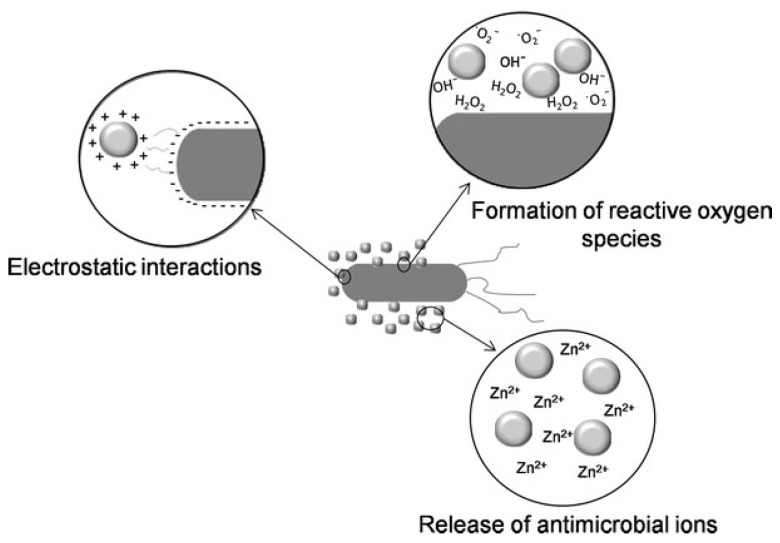
Different mechanisms of antimicrobial activity of ZnO [129].

Reactive oxygen species (ROS) production is one of nanomaterials’ primary antibacterial mechanisms. Some nanomaterials, such as AgNPs and ZnO NPs, can produce ROS when they come into contact with bacterial cells [122,123]. These organisms cause oxidative stress in the bacterial cells, which harms the lipids, proteins, and nucleic acids that make up the cells [124]. AgNPs’ positive surface charge makes it easier for them to attach to negatively charged bacterial cell walls, which in turn causes the formation of ROS and stops microbial development [125,126]. In addition to producing ROS, ZnO NPs also involved hydroxyl and singlet oxygen radicals, which enhanced their antifungal action against *Candida albicans* [127,128]. Different mechanisms of antimicrobial activity of ZnO are shown in Figure 3 [129]. The formation of ROS by nanomaterials can be influenced by the size and surface characteristics of NPs; smaller NPs often produce more ROS [130,131].

### 5.2. Ions Release

When certain metal-based nanomaterials come into contact with bacterial cells, they can release metal ions, which show antibacterial properties [132]. These ions have the ability to directly interact with the functional groups of nucleic acids and proteins, changing their structure and interfering with important biological functions. By releasing silver and palladium ions, respectively, silver nanowires and palladium nanolayers demonstrate antibacterial capabilities [133].

### 5.3. Cellular Penetration and Membrane Disruption of NPs

By changing the cell membrane’s permeability and integrity, nanomaterials can physically penetrate bacterial cells using this method, as actually carried out [134]. NPs, bound to the cell surface, change the negative charge of the cell wall and cause depolarization; such ruptures are often achieved in this way. Thus, the cell wall becomes porous, allowing the NPs to enter and destroy its integrity.

Cell death may result from this, along with cell wall collapse and component leaking [135].

### 5.4. Nano-Knife Mechanism of Graphene-Based Materials

The “nano-knife” mechanism is a special antibacterial mechanism displayed by graphene-based materials (GMs), such as graphene oxide and graphene nanosheets [136,137]. These substances have sharp nanoscale edges that can pierce and damage bacterial cell membranes like real blades would. The cytoplasmic contents of bacterial cells, such as DNA or RNA, may spill out when GMs engage with the cells because of their sharp edges’ ease of puncturing and slicing through the membrane. Cell death results from this physical damage to the cell membrane. The angle and orientation of the GMs affect this mechanism’s effectiveness; perpendicular edges penetrate the cell membrane more readily than parallel ones [136,137].

## 6. Effects of NPs on Coating Properties

The integrity of polymer films in packaging applications depends on their mechanical characteristics, such as tensile strength and elongation at break [138]. The addition of NPs can considerably affect polymer films’ mechanical characteristics. Due to their small size and high specific surface area, NPs impact the film matrix’s interfacial strength and dispersion [7].

The surface roughness of polymers can be considerably changed by adding nano-particles. The addition of the functionalized SiO_2_ particles promotes additional hydrophobization of the surface and also produces a robust dual-size rough surface [139]. Nanomaterials can significantly increase the surface roughness and reactive surface area of polymeric coatings, creating physical barriers that impede microbial adhesion. The high surface area-to-volume ratio of NPs introduces nanoscale irregularities that disrupt the surface architecture required for stable microbial attachment, thereby preventing biofilm formation [140]. The increased roughness and high reactivity of the surface also enhance the efficacy of antimicrobial action, making these coatings more effective in environments where cleanliness and sterility are critical. AgNPs can increase hydrophilicity when added to polyvinyl alcohol (PVA) films [141]; on the other hand, depending on how they are functionalized and dispersed, other NPs, such as carbon nanotubes, may increase hydrophobicity [142]. The kind and quantity of NPs added to polymers can modify their surface energy. Hydrophilic bacteria can adhere more readily to surfaces with higher surface energy, whereas surfaces with lower surface energy can prevent such adherence. Under some circumstances, TiO_2_ NPs can raise the surface energy, increasing the surface’s susceptibility to bacterial adhesion [143].

The concentration of NPs added has a significant influence on the mechanical properties of nanocomposites. However, many studies have shown that an addition of only 5% by weight of particles for nanocomposites is already enough to make the properties of a polymer do better [144,145]. It has also been shown by several researchers that although an increasing concentration of nanoparticles will enhance the modulus and tensile load-bearing force of the nanocomposites, this is at the expense of their fracture elongation rate [146]. Thus, the highly rigid NPs and their affinity for the polymer at the interface, or strong interfacial interactions, are responsible for improving the nanocomposites’ mechanical and thermal properties. This results in a highly rigid nanocomposite material with improved mechanical and thermal properties [1].

Through mechanisms like stress transmission and creating covalent and hydrogen bonds with the polymer matrix, NPs can improve the tensile strength of polymer films [147,148]. Additionally, they can fill the open gaps between polymer chains, strengthening the matrix’s intermolecular attraction forces. It has been demonstrated that various NPs, including cellulose nanocrystals, nanoclays, and graphene oxide, increase the tensile strength of biopolymer films. However, with the addition of nanoparticles, such as in the case of biopolymer film elongation breaking, the flexibility of these films will decrease [149]. Yet, because the formation of agglomerates, unevenly distributed ingredients, and loss of mechanical properties are all possible once the NP amount has been reached to exceed that level, the perfect concentration of them is important [150]. Moreover, the mechanical properties of the resulting nanocomposite films are strongly determined by the type of nanoparticle and the used biopolymer. Hydrogen bonding, interfacial interactions, and the size and form of NPs have significant impacts.

A crucial functional characteristic of polymer films is water resistance, measured using various criteria, including water solubility, swelling intensity, water content, and water vapor permeability. It has been noted that polymer films lose some water solubility when NPs are added. The particles’ ratio of dimensions and crystalline areas is responsible for this decline. NPs with larger dimensions and crystalline areas tend to decrease the water solubility of the films [151,152]. The type of NPs used also influences the solubility of the film. NPs with very low solubility compared to the polymer chains can decrease the hydrophilicity of the polymer matrix, resulting in a decrease in solubility [153]. The use of NPs may reduce the water content of polymer films. The interaction between the functional groups of the biopolymer chain and the NPs is considered the cause of this phenomenon. As a result of the interaction between polymer chains and NPs, the matrix’s accessible spaces may be reduced, which could lower the water content [151]. By reducing water solubility and decreasing water content, the addition of NPs can enhance the water resistance of polymer films. These properties are crucial for applications where water resistance is desired, such as food packaging, coating materials, and barrier films.

Water vapor permeability is a critical property for films used in packaging, especially for food products where moisture control is essential. The addition of NPs to polymer films can affect their water vapor barrier properties. NPs, particularly impermeable ones, can hinder water vapor diffusion through film by impeding the mobility of polymer chains and creating a tortuous path [154].

However, the presence of NPs can decrease the permeability of water vapor which provides a good opportunity for using them as materials that only need very short cure times. Nevertheless, it is important to add that surpassing the critical concentration of NPs may allow water vapor to permeate nanocomposite films. Films can have poor barrier properties as they reach high levels of nanoparticle loading. Therefore, before a significant negative effect on water vapor permeability occurs, the critical concentration refers to the point when the benefits of introducing NPs and reducing their permeability are more than canceled out. Therefore, carefully engineering and controlling the concentration of NPs is a necessary requirement in order to achieve the barrier properties for water vapor that are desired in nanocomposite films. In addition to modifying the physical and chemical characteristics of the particle, modulating its concentration is also critical for realizing performance-enhancing barrier materials without compromising performance.

By preventing the growth of bacteria, NPs boost the antimicrobial activity of polymer-based films, which is crucial for guaranteeing food safety and extended storage stability. NPs can exhibit bactericidal (killing) or bacteriostatic (inhibiting growth) effects on microorganisms, depending on their type and interaction with bacterial cells. Gram-negative bacteria are generally more sensitive to NPs than Gram-positive bacteria due to differences in their cell wall structure [7]. The specific interaction between NPs and bacterial cells can vary depending on the characteristics of the NPs and the target microorganisms. Research findings indicate that polymers coated with composites of NPs can sustain their antibacterial effectiveness for prolonged periods of time. ZnO NPs may show selective toxicity, making them more effective against some bacterial strains than others; however, AgNPs are very efficient against a wide range of bacteria and fungi [155,156,157].

**Figure 4:**
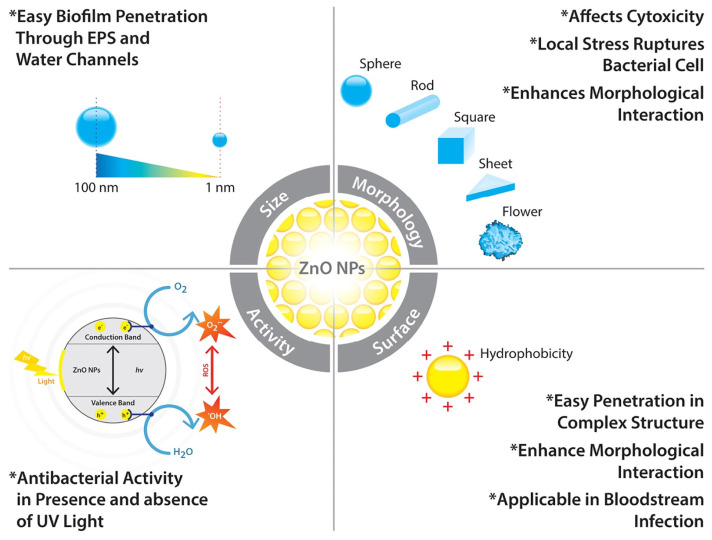
Role of size, shape, and interior properties of ZnO in Enhancing Coating Effectiveness Against Biofilm-Related Infections [159].

Nanomaterials not only inhibit initial microbial adhesion but also disrupt the structure of biofilms if microorganisms manage to adhere. The physical roughness and chemical reactivity of nanomaterial-enhanced coatings prevent stable biofilm formation, which is typically resistant to conventional cleaning and antimicrobial treatments [158]. For instance, GO physically disrupts microbial cell membranes, while the reactive species generated by TiO_2_ NPs and ZnO NPs can penetrate and kill microorganisms within biofilms, weakening their structure and making them easier to remove [28]. Size and shape, surface and interior properties of ZnO NPs can enhance the properties of the coatings in biofilm-related infections as shown in Figure 4 [159].

The physiological properties of NPs play a crucial role in their antimicrobial activity. In antimicrobial activity, the size of NPs is a key factor, and the smaller they are, the better [160]. Smaller NPs have their own benefits. When they invade the microbial cells, they can penetrate better. The result is that more ROS reach these cells more easily. Because the dissolution rate is higher and they provide a larger surface-to-volume ratio—all of these point toward their increased antimicrobial activities. However, there have been cases in which larger NPs likewise have been successful as agents for killing bacteria and other germs [161]. By appropriately adjusting their size, concentration, and other physicochemical properties, desired antimicrobial activities can be achieved.

The shape of NPs also influences antimicrobial activity, with different shapes causing variable cellular damage. For example, cuboidal-shaped NPs showed more antibacterial activity than nanocubes and rods exhibited [162]. The roughness of NPs has been less studied but has been found to affect bacterial protein adsorption and adhesion [163]. The charge of NPs is another critical factor, with cationic NPs showing enhanced antimicrobial activity due to their electrostatic attraction to negatively charged bacterial cell walls and increased ROS production [164].

Using a simple one-pot method, Kang et al. [51] created cellulose nanocrystal/zinc oxide (CNC/ZnO) nanohybrids with reinforcing and antibacterial capabilities. With increasing CNC/ZnO nanoparticle concentration up to 10 weight percent, the Young’s modulus and tensile strength of nanocomposite films gradually improved. The 10% CNC/ZnO composites had *E. coli* and *S. aureus* inhibition rates above 88.8%. Yakdoumi et al. [164] found that, in comparison to pure PLA, the Young’s modulus of PLA/TiO_2_-PDA-MWCNTs and PLA/PDA-MWCNTs was enhanced by 161% and 113%, respectively, while the hardness was increased by 815% and 79%, respectively. Additionally, modified MWCNTs nanocomposite films showed higher antibacterial and antifungal activities than pure PLA.

In order to create nano-biocomposite films, Dairi et al. [44] combined plasticized cellulose acetate/triethyl citrate (CA/TEC) with gelatin-modified montmorillonite nanoparticle, AgNPs, and thymol (Th). The addition of clay slightly raised the glass transition temperature of CA. The only other factor that improved CA’s thermal stability was the addition of clay. While adding more of both chemicals to films reduced their optical clarity, they significantly improved their UV barrier properties. While Th started an antagonistic effect, the clay enhanced the tensile strength and oxygen barrier characteristics. With the help of potato peel, Min et al. [165] created CDs, which significantly improved the gelatin film’s water vapor permeability (by 28%) and hydrophobicity (by 9% and 13%) without significantly altering its mechanical qualities.

The introduction of nanomaterials can alter the hydrophobic or hydrophilic nature of the coating surface, impacting microbial adhesion. Nanomaterials that increase hydrophobicity can create surfaces that repel water and certain microorganisms that prefer hydrophilic environments [166]. Conversely, hydrophilic surfaces can form a hydration layer that prevents close contact between the surface and microorganisms, thereby reducing adhesion [167]. Positively charged surfaces can attract negatively charged microbial cell walls, leading to cell rupture and death, while negatively charged surfaces can repel similarly charged microbial cells, reducing adhesion.

Tammina and Rhim [168] incorporated nitrogen-doped CDs, derived from polyethylene glycol, into a functional film made of carboxymethylcellulose (CMC) and agar. Adding CDs, especially nitrogen-doped CDs, to CMC/agar-based films led to decreased mechanical strength and hydrophobicity on the film surface. However, the water vapor barrier properties remained the same, while the film exhibited enhanced UV blocking, antioxidant, and antibacterial properties. Hosseinzadeh et al. [73] functionalized magnetic GO with crystalline nanocellulose and zwitterionic polymers to improve the properties of membranes. This functionalization resulted in membranes with a more hydrophilic surface and lower roughness. The composite nanofiltration membrane, after modification, showed enhanced pure water flux, flux recovery ratio, and anti-fouling capability. The pure water flux increased significantly at 2 bar pressure, from 61.18 LMH (liters per square meter hour) in the original membrane to 123.3 LMH in the nanocomposite membrane. A major strength of these studies is their demonstration of the potential being offered in functional films for nanomaterials to modify their properties. Nitrogen-doped CDs in CMC/agar-based films increased particular properties such as UVA and UVB resistance, anti-oxidation, and antibacterial capabilities. Meanwhile nanocellulose-functionalized magnetic GO films formed membranes and zwitterionic polymerizations with low antifouling rates, and higher water flux than the original film. These findings are expected to open up a wealth of possibilities for nanocomposite films, including applications in areas such as packaging, water treatment, and membrane technology.

Koshy et al. [45] used soy protein isolate films embedded with AgNP to bind carbon dots and chitin nanowhiskers. It was found that the combination of CNW and AgNP considerably enhanced the mechanical strength and thermal stability of soy protein isolate film and lowered the moisture content. Waterborne epoxy coatings can be made more resistant to corrosion and fouling by adding effective NPs such as graphene oxide, amino-silane functionalized ZnO quantum dots, and their nanohybrids (F-GO@ZnO QDs) [169]. The findings show that the steel substrates’ highest barrier and corrosion resistance could be achieved using the uniformly dispersed F-GO@ZnO QDs in the coating’s fracture surface. The nanocomposite coatings, on the other hand, have higher water contact angles and more vital substrate adherence.

The hydrogels made by Cui et al. [170] using lignin nanoparticles as reinforcement revealed excellent mechanical properties, including exceptional durability, long-term adhesiveness, and quick and effective self-healing. With good compression strength of 810 kPa and elasticity (stretching to 13 times its initial length), NPs substantially enhanced the hydrogels. The presence of metal ions, lignin, and Ag NPs gave the composite hydrogel exceptional conductivity, ultraviolet-blocking capabilities, and significant antibacterial activity. A green, high-performance soybean meal-based glue with an improved residual ratio of 85.8% and a decreased moisture absorption of 16.5% was created by Cao et al. [171] using a bioinspired mineral–organic hybridization method. Hydroxyapatite and the tannic acid complex also reduced heat transfer and snuffed out oxygen-free radicals, improving the adhesives’ ability to withstand flames.

## 7. Applications of Nanoparticle-Enhanced Coatings

**Figure 5:**
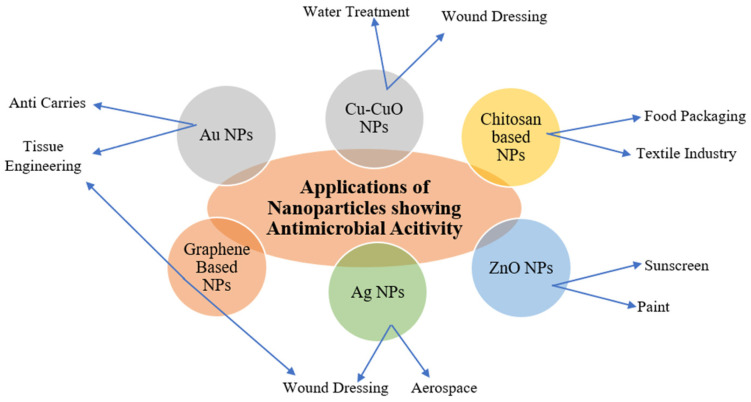
Applications of nanoparticles.

NPs have several applications in sectors including healthcare, biomedical, textile, food technology, etc. as shown in Figure 5.

### 7.1. Healthcare Sector

ZnO is utilized in dentistry as a temporary filling material and as part of dental pastes. Additionally, ZnO is an excellent medium in sun creams since it absorbs UV rays, is gentle on the skin, and is quickly absorbed [172]. The application of ZnO as an antibacterial ingredient in paints, mouthwashes, and surface coatings to inhibit biofilm establishment has also been researched [155].

**Figure 6:**
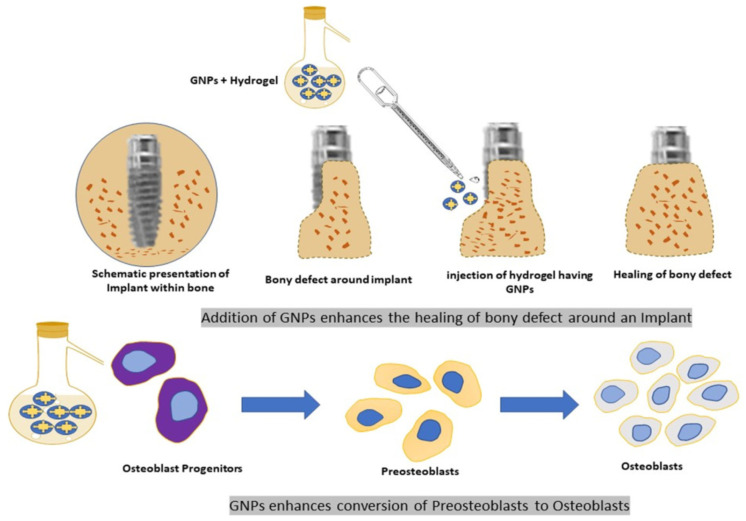
AuNP acts as an osteogenic agent for bone regeneration (with permission) [51].

Due to their nano size, AuNPs have a higher surface area and can readily interact with both inorganic and organic molecules. Because of this trait, they are possible agents (anticaries) for avoiding tooth decay. According to Yi et al. [173], AuNPs also have strong surface specificity and biocompatibility, making them suitable for application as osteogenic agents in bone regeneration, as shown in Figure 6. On titanium implant surfaces, chitosan gold NPs (Ch-AuNPs-PPARc) have been tested for their capacity to reduce inflammation and encourage osteoblast growth. According to Bhattarai et al. [174], these coated implants function as gene-activated materials that improve biocompatibility by boosting gene transfection and enabling protein and cell adhesion. Dental adhesives’ flexural and tensile strength have also been proven to be improved by the inclusion of gold nanoparticle particles [175].

Graphene-based chitosan fibers can be employed directly in wound healing since they are antimicrobial [72]. Due to their low toxic qualities, NaAlg/GO hydrogel fibers, generated from their equivalent fibers, have future uses in wound-dressing materials [176]. Graphene-based materials, such as GO and G, have shown potential in various biomedical applications, including wound healing, tissue engineering, and drug delivery [73]. These materials possess unique properties that make them suitable for such applications, such as excellent mechanical properties, large surface area, and biocompatibility. Hydrogels crosslinked with silver (Ag) and graphene (G) mixtures have been investigated for their potential in wound healing. In one study, hydrogels were crosslinked with Ag/G mixtures using acrylic acid and N, N_0_-methylene bisacrylamide [177].

High swelling capacity, antimicrobial abilities, biocompatibility, and improved mechanical properties are all desired properties in a hydrogel possessing this Ag: G ratio of 5:1. These properties are crucial for promoting faster wound healing and providing an environment conducive to tissue regeneration. Hydrogels made from carbon-based materials that incorporate graphene offer some advanced possibilities. The hydrogels can, therefore, acquire antimicrobial properties, better strength (toughness), and biocompatibility by adding silver and a small amount of graphene; all of these factors are vital for effective wound healing.

PU/siloxane/GO networks demonstrated excellent antimicrobial effects and enhanced wound-healing processes [178]. G-based nanocomposites in tissue engineering promoted biocompatibility, mechanical stability, and favorable properties for bone regeneration; by combining GO with chitosan, a 3D scaffold was created, which displayed excellent biocompatibility, enhanced mechanical characteristics, pore formation, and bioactivity [179].

Numerous biotechnological applications, including antibacterial paint and coating for use in biomedical/hospital, domestic, and aerospace settings, can benefit from the regulated release of Ag^+^ from polymer/metal nanocomposites [180]. Additionally, compared to any constituent phase, the biopolymer chitosan/coated Ag nanocomposite showed more excellent antibacterial activity against two strains of *S. aureus* [181]. Nanocomposite films based on chitosan/poly(vinyl pyrrolidone) (PVP)/nanocellulose exhibited compatibility, low cytotoxicity, and desirable properties for wound dressings [182]. Chitosan films containing 5% chlorhexidine (CLX) on a montmorillonite (MMT) matrix showed non-cytotoxicity, suggesting localized and prolonged CLX release for wound dressings [183]. Flexible nanocomposite carboxymethyl cellulose (CMC) hydrogel films with graphene quantum dots and the anticancer drug doxorubicin demonstrated pH-sensitivity, prolonged drug release, and low toxicity against blood cancer cells [184]. Chitosan films with SeNPs produced electrically conductive cardiac patches, offering antioxidant activity, and potential for cardiac tissue engineering [185].

In the Mg-Zn (MZ) alloy matrix, graphene nanoplatelets (GNPs) and carbon nanotubes (CNTs) nanosystems were synthesized by Liu et al. [186] as reinforcements. The study shows that MZ/GNPs + CNT composites can be used as a new generation of implants and treatment materials for bone infections, including *S. aureus* and *E. coli* growth. PMMA nanocomposites were synthesized using hydroxyapatite nanofibers and magnesium phosphate nanosheets. These materials possess not only mechanical characteristics but also bioactivity, and cytocompatibility that are superior to traditional bone cement composites. They could introduce a new paradigm in designing future bone cement composites due to their superior bioactivity, mechanical characteristics, and cytocompatibility [187].

### 7.2. Food Packaging Industry

The development of anti-microbial food packaging materials with NPs brings additional benefits like mechanical properties, thermal stability, and the ability of materials to keep pollutants, gasses, and moisture out. In particular, the packaging with embedded NPs can even restrict many kinds of microbial growth into finished food products. This prolongs their shelf life. By adding NPs to a biodegradable composite film, it becomes possible to adjust packaging materials for good stretchability and fresh-keeping properties while featuring other desirable qualities [188].

Due to their outstanding tensile strength and antibacterial qualities, graphene-based materials, such as GO, show potential for food packaging applications [73]. Films used for packaging are made lighter and more durable by adding graphene [189]. The environmentally friendly process of creating chitosan/GO nanocomposites results in materials with strong mechanical and barrier properties that prevent bacterial development while retaining qualities needed for food packaging [190,191]. Polylactic acid (PLA)-based antimicrobial polymeric films with clove essential oil and GO show increased flexibility, oxygen permeability, and antibacterial activity against *S. aureus* and *E. coli* [167]. Nanocomposite hydrogels with improved thermostability, tensile strength, modulus, and antibacterial activity against various bacteria and fungi are made when mixed with chitosan hydrogel and iron oxide-coated GO [192].

AgNPs were successfully added to an HPMC matrix to improve the material’s mechanical, barrier, and antibacterial properties in food packaging [193]. The migration of silver in a PLA/silver-based nano clay composite for food packaging was examined in a different study, and it was found to be below permissible limits established by the European Food Safety Agency (EFSA). The antibacterial activity of this composite supports its potential use in food packaging to increase food quality and safety [194].

Shahabi et al. [195] studied the effect of halloysite nanotubes in soy protein isolated/basil seed gum film activated with propolis. Following the encapsulation of propolis, the antibacterial properties of the film were considerably improved. The developed films demonstrated practical efficiency for use in food packaging systems. In order to create high-performance and multifunctional CMC-based intelligent, active films, Tang et al. [196] effectively synthesized cobalt-based metal–organic framework (Co-MOF) nanosheets with ammonia-sensitive and antibacterial activities. Successfully developed CMC/Co-MOF nanocomposite films have a wide range of potential uses in intelligent, active packaging.

In order to create multipurpose food packaging materials, Hu et al. [53] synthesized biodegradable polyvinyl alcohol/starch (PVA/ST) films compatible with rod-like ZnO NPs. The produced PVA/ST/ZnO films were tested to stop microbiological contamination and increase the shelf life of freshly cut carrot slices. These findings suggested that the highly transparent and multifunctional PVA/ST/ZnO nanocomposite films had a wide range of potential applications in active food packaging. The R-cellulose/metallic NP hybrids demonstrated intense antibacterial activity against *E. coli* and *L. monocytogenes.* They were synthesized by Shankar et al. [197] as antimicrobial hybrid nanomaterials of Ag, CuO, or ZnO NPs during the regeneration of cellulose from cotton linter and microcrystalline cellulose.

High antioxidants (DPPH 12.7% and ABTS 67%) and robust antibacterial action against damaged cells were demonstrated by nitrogen-doped carbon dots. *E. coli* and *L. monocytogenes* are anticipated to be incorporated into food packaging films to develop sustainable multifunctional food packaging materials [168]. To meet the rising need for food safety, Feng et al. [198] synthesized intelligent, active packaging films with ammonia-sensitive performance to retain and monitor the freshness of meat meals. In order to create sodium alginate (SA)-based films, cobalt-based metal–organic framework (Co-MOF) NPs with ammonia-sensitive and antibacterial properties were first created. The PBST/MgO/Ag nanocomposite films created by Zhang et al. [199] showed better antibacterial properties against *Salmonella paratyphi B*, *E. coli*, and *S. aureus* than poly(butylene succinate-co-terephthalate) coatings. Experiments on cherry tomatoes demonstrated how well the films may be preserved.

When used in a Fenugreek gum matrix, sodium Mt. halloysite NPs are effective as an antibacterial in food packaging. They demonstrate antimicrobial activity against bacteria such as *L. monocytogenes*, *S. aureus*, *B. cereus*, and *E. coli*. Additionally, they improve the packaging materials’ tensile strength and oxygen barrier qualities, resulting in improved food safety and quality [200]. Orange peel extract and Cloisite 30B are added to whey protein isolate–gelatin films to improve their tensile strength, flexibility, and optical qualities. These movies specifically display excellent antibacterial efficacy against Gram-negative bacteria like *E. coli*. They contribute to the safety and preservation of food by being suited for long-term usage as antibacterial food packaging materials [201].

Liu et al. [186] synthesized graphene nanoplatelets (GNPs) and carbon nanotubes (CNTs) and incorporated them into the Mg-Zn (MZ) alloy matrix as reinforcements.

The original aim was to study how the incorporation of GNPs + CNTs affected the infiltration and growth of bacteria—particularly *S. aureus* and *E. coli*. These remarkable composites developed MZ/GNPs + CNTS and managed to reduce the infiltration and growth of *S. aureus* and *E. coli*. These coatings can be used for an implant in the bone and related treatments. Overall, the study supports the potential use of MZ/GNPs + CNT composites as practical materials for implants and the treatment of bone infections due to their ability to reduce bacterial infiltration and growth.

### 7.3. Textile Industry

ZnO nanostructures have gained much popularity as UV-resistant textile coatings. They are beneficial for preventing unwanted stains on clothing because of their self-cleaning and water-repellent properties. ZnO NPs have been produced utilizing various techniques, including hydrothermal growth on cotton fabrics coated with SiO_2_ or deposition on cotton and wool fabrics, to create UV-protective textiles [202].

The textile industry has developed uses for graphene–polymeric nanocomposites, with advantages like enhanced antibacterial activity, mechanical strength, conductivity, flame resistance, UV protection, and gas barrier qualities. Both Gram-positive and Gram-negative bacteria have been significantly inhibited by fabrics containing GO or reduced graphene oxide (rGO) [203]. With GO or rGO, cotton and cotton/nylon textiles showed high rates of bacterial inhibition [204]. When synthetic fabrics like polyester were treated with rGO/Ag nanocomposites or GO/PVA, bacterial growth was effectively inhibited [205].

A coating of alkoxy silanes, fluoropolymer, silane quaternary ammonium salt, and silica NPs was applied to silk fabric using a potato dextrose agar medium. The coating’s superhydrophobic properties further boosted the strong antibacterial properties of the coated silk fabric. This can be utilized to make multipurpose coatings for the textile sector [206]. A 100% antibacterial activity against *E. coli* was obtained when AgNP and an AgNP/reduced graphene oxide nanocomposite were used on polyviscose cloth. The antibacterial activity dropped to 90% after 12 regular washing cycles. The coating showed rinse–reuse properties, making it suitable for medical textiles that must be used again and washed [207].

Even at low ZnO concentrations, applying hybrid ZnO/Chitosan NPs on cotton fabric demonstrated increased antibacterial activity against both *S. aureus* and *E. coli.*

The bactericidal action was perennial after repeated washings, all showing its long-term effectiveness. Moreover, chitosan-based coatings have increased biocompatibility which makes them ideal for the use in textile industry. Poly(diallyldimethylammonium chloride)/Poly(methacrylic acid-capped silver NPs) on nylon and silk fibers can deliver significantly reduced levels of *S. aureus* bacteria. On silk fiber, antibacterial activity was reduced by 80%. And on nylon fiber, its antimicrobial activity was reduced by 50%. Potential uses for this coating include the creation of antibacterial fabrics and water sanitation [208].

### 7.4. Other Applications

**Figure 7:**
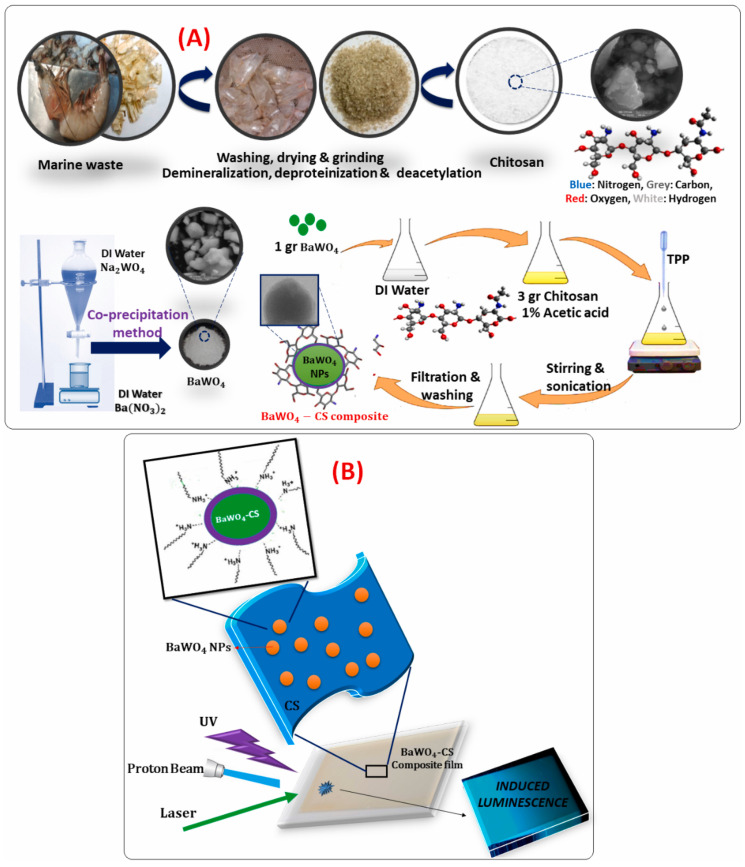
(**A**) Brief diagram of experimental works for preparation of samples and (**B**) Graphical scheme of the hybrid BaWO_4_-CS nanocomposite (with permission) [209].

Studies have been reported on using flexible, wearable, and self-powered photoelectronic devices in communication, wearable electronics, healthcare, infrastructure monitoring, alternative energy sources, and fire monitoring. Due to their non-toxic nature and advantageous physical and chemical properties, tungstate groups coupled with biopolymers, particularly chitosan, provide hope in this field. Different substrates can incorporate these materials, including metal, glass, silicon, sapphire, flexible plastics, and polymers. In order to build flexible optical sensors, a study used nanostructured barium tungstate (BaWO_4_) particles as NPs in a chitosan biopolymer matrix [209]. The synthesis is shown in Figure 7. No harmful solvents or surfactants were utilized during the drying process at room temperature, which produced the NPs by the co-precipitation method. The resulting sensors demonstrated potential for numerous uses.

The innovative, highly antifouling composite membranes were created using the phase inversion procedure by mixing the antibacterial copper oxide (CuO) and strong hydrophilic GO with poly (vinylidene fluoride) (PVDF). Notably, the membrane shows an effective antibacterial capability. The composite membranes showed remarkable antifouling performance thanks to the hydrophilic and antibacterial membrane surface. They exhibit enormous promise for wastewater treatment [210]. An elastomeric nanocomposite with antibacterial and antifungal properties has been created. It comprises a rubber blend matrix and Nanobent^®^ ZR2 (a modified bentonite clay nanoparticle). They are sought-after in biomedical engineering, medicine, and the food sector due to their strong antibacterial and antifungal properties [65].

**Figure 8:**
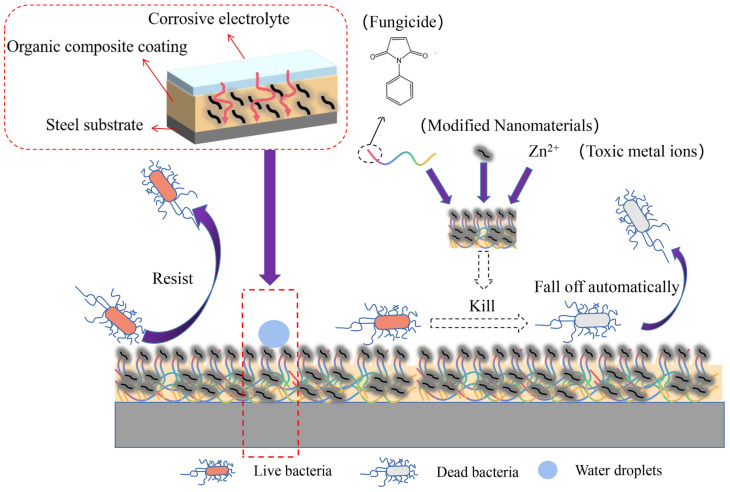
Schematic diagram of the antibacterial and anticorrosive mechanism of composite coating (with permission) [211].

A fluorinated silica-coated hydroxylated multi-walled carbon nanotubes nanocomposite (F-MWCNTs-OH@SiO_2_) was disseminated in PAZ/N-PMI resin to manufacture the composite coating samples. The antibacterial rate of the composite coating was found to be >99.98% against *S. aureus* and *E. coli.* The mechanism of antimicrobial and anticorrosive activity is shown in Figure 8. This study offers various potential applications for creating superior maritime anti-corrosion and antifouling organic coatings [211].

ZnO and multi-walled carbon nanotube (MWCNT) were used as dual NPs in a polyethersulfone (PES) membrane that Pang et al. assessed for performance and antifouling qualities. The membrane exhibits effective antifouling capabilities, lower relative flux reduction (RFR), and notable antibacterial characteristics. The research’s practical uses are primarily in river water recovery [212]. Ismail et al. created a brand-new quaternized polydopamine-anchored reduced graphene oxide (QSiPD-rGO) nanohybrid. The hybrid membranes demonstrated exceptional antibacterial activity (against *E. coli*) and excellent fouling resistance. This has significant applications in wastewater treatment [53].

## 8. Challenges and Future Perspectives

### 8.1. Challenges

The broad range of physical and chemical properties of NPs in polymeric coatings necessitates standard test methods and better characterization. Standard evaluation methods are required for better characterization and antibacterial efficacy, toxicity, and mechanical properties. NPs in food technology must be assessed for toxicity to ensure safety. The evaluation methods such as in vitro and in vivo studies, genotoxicity tests, and hemocompatibility tests are required to check the toxicity of the NPs in use. Oxygen and moisture permeability tests should be conducted to check the barrier properties. Thermal analysis should be carried out to test the stability under high temperature and food storage conditions. Leaching tests and abrasion resistance tests should be performed to test the stability of these NPs in the textile industry.

Because of the complicated impacts of NP synthesis procedures, NP agglomeration, NP confounding of routine microbiology assays, and interaction of NPs with cells and media elements, determining NP toxicity and dosage for actively developing bacteria is extremely difficult. According to Kadiyala et al. [213,214], individual NP mass can be computed using the density of the NP material and the volume of the NP shape. A standard model of NP toxicity toward bacterial cells might be created by disclosing the number of beginning cells employed, the number of possible particles and NP aggregates exposed to these cells, and the exposure duration. Since many NPs may combine or agglomerate and are likely to produce protein coronas in the growth media, it can be difficult to report the precise amount of NPs in contact with bacterial cells. These NPs’ solubility varies with time and is a significant factor in their toxicity. Additionally, when evaluating the antibacterial mechanism and dose-response of NPs, a number of aspects should be taken into account. Variations in the amount of cell death or inhibition, the types of bacterial species used, the initial number of cells exposed to NP, and the techniques used to prepare NPs and their suspensions can cast doubt on the literature’s findings that the investigated NPs are effective antibacterial agents. To check whether NPs are effective or not in accordance with a standard toxicity evaluation, ongoing testing is required. In order to provide better substitutes for antibiotics and disinfectants in biomedical applications, more research needs to be carried out to better understand how NP works against bacteria.

The usage of nanotechnology in polymeric coatings has raised concerns from both consumers and regulators. High production costs, strict safety regulations, and fears about damage to human health and the environment are part of what stands in the way of NP-enhanced coatings. Because nanocomposites often combine with tests, both oxidize and get contaminated, though nanoparticle handling and processing continue to create challenges. Because NPs often combine, oxidize, and become contaminated, handling and processing nanocomposites still offers challenges. The adverse effects and biological responses to NPs vary depending on their physical and chemical properties, as well as experimental conditions.

When applied to coatings and nanomaterials, characterization techniques also have significant limitations and difficulties. Because coatings have complex compositions, it can occasionally be challenging to accurately identify the functional group by FTIR and explain the overlapping peaks. Because of interference, volatile solvents can produce noise and complex spectra. Environmental factors that affect coating stability and can produce varying FTIR results for each batch are also taken into consideration [215,216]. Complexity in the coatings even leads to poor diffraction patterns in XRD analysis. Rough surfaces and non-uniform coatings can give poor diffraction data/patterns. The surface can reduce the accuracy, scatter the peaks, and make it difficult to identify the data. Some NPs may exhibit low crystallinity, which can give weak diffraction patterns that may hinder the detection of crystalline phrases. Multilayer coatings contain additives, metal ions, NPs, solvents, etc. which can give a complex background with noise [217,218,219]. In TEM and SEM analysis, the use of a high-energy electron beam can distort the structure resulting in alteration in the functionality of the NPs. Beam cam elevates the temperature, and causes thermal degradation of the materials, especially in poorly conducting samples. Atomic displacement can damage the crystalline structures of the NPS with low molecular weight. This analysis can even cause sputtering which can result in loss of material and defect formation. Sputtering can also interfere with the surface morphology as it targets the surface atoms. The coatings that are non-conducting can accumulate charge due to electron beams which can result in poor imaging and analysis [220].

Improved characterization using standardized testing techniques is necessary to ascertain the ultimate destiny of NPs and their potential detrimental or inflammatory impacts on organisms. As a result, more reactive oxygen species are able to more readily enter these cells. They appear to have stronger antibacterial properties because of their greater surface-to-volume ratio and faster rate of breakdown. With the rapid development of nanotechnology, it is important to consider any adverse effects associated with discharge into the environment. It must be determined whether AuNPs will enter the bloodstream through skin, inhalation, or ingestion after humans come into contact with them.

### 8.2. Problems Associated with the Usage of Nanoparticles

**Figure 9:**
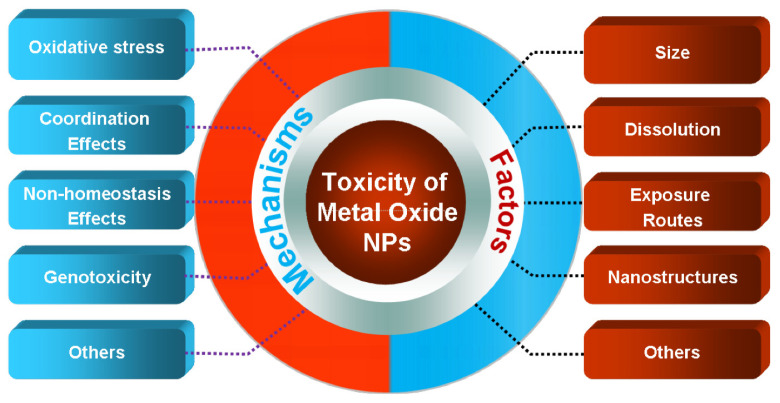
Some toxic effects of CuO and ZnO NPs and their key factors [17].

NPs pose a brand-new toxicological problem. The production of reactive oxygen species, protein misfolding, membrane disruption, and direct physical harm are some of the hypothesized mechanisms of toxicological damage that have been discovered. Nanoparticles have the ability to directly or indirectly impact membrane stability, which can result in cell death [221]. The toxic effect of ZnO and CuO nanoparticles and their factors are shown in Figure 9.

Li et al. [222] used the Comet, Pig, and Mouse Micronucleus assays to assess the mutagenicity and clastogenicity of AgNPs with varying sizes and coatings. According to the study, AgNPs made it to the testing tissues (liver for the Comet assay and bone marrow for the mouse and Pig-a assays). AgNPs coated with silicon and PVP both caused oxidative DNA damage in the liver of mice. After oral treatment, rats exposed to anatase TiO_2_ NPs demonstrated that the particles might cause DNA double-strand breaks in bone marrow cells [223].

The deposition of ultrafine particles in the human nose/oral region was measured by Cheng et al. [224], who discovered that nasal deposition rose as human age decreased. It was found that the outcomes matched predictions of turbulent diffusion deposition. In adult human tracheobronchial airways, Cohen et al. [225] studied the deposition of 40 nm particles and found that it was higher than what was predicted theoretically for laminar flow. In some areas, deposition was twice as high as anticipated. It has been shown that ultrafine nickel and cobalt can increase inflammation brought on by free radicals [226]. Studies showed that ultrafine particles of copper oxide and zinc oxide from coal combustors and smelters had an impact on the lungs [227]. The action was amplified by the sulfur oxide coating on the particles.

Choi et al. [228] showed that while the non-modified cadmium telluride quantum dots caused lipid peroxidation in the cells, the toxicity of quantum dots was decreased following surface modification with N-acetylcysteine. This affects biomedical applications including medication delivery, imaging molecules, and even genes [229]. The presence of the stabilizer CTAB, which even had a significant cytotoxic effect after washing, may have contributed to the cytotoxicity of gold nanorods. According to Niidome et al. [230], PEG-modified gold nanorods that had excess CTAB removed did not exhibit cytotoxicity.

The textile industry also faces some challenges when it comes to the use of NPs. The stability of the nanomaterials existing in the textile depends on its fabric binding, and the influences on the fabric in the processes including manufacturing, utilization, and disposal/recycling, which could harm the textile material or the bonding between the fibers and nanomaterials. This could happen due to mechanical stress, abrasion, temperature changes, high temperatures, detergents, solvents, water, body fluids, and ultraviolet radiation [231].

### 8.3. Future Perspectives

Subsequent developments in polymeric coatings will be focused on preventing nanoparticle aggregation through the use of advanced stabilizing methods. NPs can be maintained well-dispersed inside the polymer matrix through the application of dispersants, surface functionalization, and core–shell structure formation. To counteract contamination and oxidation, future polymeric coatings will incorporate methods to increase the stability of embedded NPs. One of the main goals for future polymeric coatings will be developing safe and biocompatible compositions. Improvements in surface chemistry and nanoparticle design will help to maintain high antibacterial activity while lowering cytotoxicity. Cytotoxicity will be reduced through advances in nanoparticle design and surface chemistry, while high antibacterial activity is preserved. At present, the future of polymeric coatings is in issues relating to biodegradability, sustainable development, etc. New types of environmentally friendly coatings can be produced by combining biodegradable polymers with biogenic or natural NPs.

The successful realization of NP-enhanced polymeric coatings will hinge on the setting up of a clear regulatory environment and sorting out problems relating to market integration. Setting up safety standards in ministry—company cooperation will greatly expedite approval and commercialization. Future studies may well concentrate on novel coating technologies. These will be seen not only in smart coatings that react to environmental states, for instance, but self-healing coatings which can repair themselves after being damaged. NPs in the atomic and molecular planes offer a promising approach to disease prevention and more advanced biological uses. At present, state-of-the-art technologies can be combined with NPs to produce dynamic, flexible protection coatings that give birth to a great many types of new applications across every industry.

## 9. Conclusions

Nanoparticle-doped antimicrobial and antifungal polymeric coatings have a wide range of applications in healthcare, food packaging, textiles, and water treatment to address microbial contamination and enhance durability. NPs enhance the functionality of these polymeric coatings in terms of mechanical strength, thermal stability, and barrier properties, in addition to their potent antimicrobial activity. Coatings doped with NPs follow different mechanisms of action that include reactive oxygen species (ROS) generation, ion release, physical disruption of microbial membranes, and biofilm inhibition. NPs such as Ag, Au, Cu, CuO, ZnO, and carbene-based materials provide functionalities including UV resistance, corrosion resistance, self-cleaning, and hydrophobic or hydrophilic surface modifications.

However, these NP-based polymeric coatings possess several challenges as well. Better characterization and uniform test procedures are required due to the wide variety of NPs’ physical and chemical characteristics in polymeric coatings. For improved characterization and antibacterial activity, toxicity, and mechanical qualities, standard evaluation techniques are needed. Problems with NP-enhanced coatings include high production costs, stringent safety requirements, and environmental and human health. When applied to coatings and nanomaterials, characterization techniques also have significant limitations and difficulties. Future research should be focused on more eco-friendly solutions and innovations, like self-healing and biodegradable materials, to enhance the functionality of these coatings. Improved characterization is needed to determine the final fate of NPs and their possible harmful or inflammatory effects on organisms, and standardized testing methods are required.

## Figures and Tables

**Table 1:** Summary table of different types of NPs.

Type of Nanoparticle	Key Properties	Antimicrobial Mechanisms	Synthesis Methods	Examples of Applications	Advantages	Limitations	References
Chitosan NPs (CNPs)	Biodegradable, non-toxic	Electrostatic interactions, nutrient binding	Ionic gelation, emulsion crosslinking	Food packaging, wound dressings	Biocompatibility, natural antimicrobial	Limited stability in specific environments	[13]
Silver NPs (AgNPs)	High surface area, ion release, conductivity	Membrane disruption, enzyme inhibition	Chemical reduction, green synthesis	Medical devices, wound dressings, textiles	Broad-spectrum activity, low concentrations	Potential cytotoxicity, high cost	[14,15,16]
Copper NPs (CuNPs)	Conductive, catalytic	Membrane damage, oxidative stress	Chemical reduction, thermal decomposition	Antimicrobial coatings, paints, fabrics	Cost-effective, strong antibacterial properties	Oxidation and stability issues	[17,18,19]
Zinc Oxide NPs (ZnO-NPs)	Photocatalytic, UV protection	ROS generation, membrane damage	Sol–gel, hydrothermal, vapor-phase	Sunscreens, coatings, food packaging	Effective under UV, low toxicity	Limited activity in the dark, aggregation	[20,21,22]
Titanium Dioxide NPs (TiO_2_-NPs)	Photocatalytic, UV-protective	ROS generation, photocatalysis	Sol–gel, hydrothermal, precipitation	Coatings, self-cleaning surfaces	Effective under UV light, stable	Limited activity without UV, potential photocatalytic toxicity	[23,24]
Silica NPs (SiO_2_-NPs)	High surface area, functionalizable	Physical barrier, ROS generation	Stöber method, sol–gel	Coatings, composites, biomedical applications	Chemical versatility, ease of functionalization	Limited direct antimicrobial activity often requires functionalization	[25]
Copper Oxide NPs (CuO-NPs)	Photocatalytic, high stability	ROS generation, membrane damage	Sol–gel, precipitation, thermal decomposition	Paints, coatings, textiles	Strong antimicrobial properties, cost-effective	Potential toxicity, dark brown color limiting esthetic applications	[26]
Graphene Oxide (GO)	Large surface area, high conductivity	Physical disruption, oxidative stress	Hummers method, thermal exfoliation	Water treatment, coatings, electronics	High efficiency, stability	Potential environmental impact, cost	[27,28]
Carbon Nanotubes (CNTs)	High strength, electrical properties	Physical damage to cell walls, ROS generation	Arc discharge, chemical vapor deposition	Biosensors, filtration systems	High mechanical properties, effective at low concentrations	High cost, potential toxicity	[29,30,31]

## Data Availability

Not applicable.
